# Registered Practitioners

**Published:** 1881-04

**Authors:** 


### REGISTERED PRACTITIONERS.

The following additional names have been registered in the County Clerk's office. This, in addition to those furnished in our previous numbers, gives a complete record of registered physicians to this date.

| Names. | Graduate of | Dates. |
|---|---|---|
| Andrews, Judson B. | Medical Department of Yale College | Jan. 15, 1863. |
| Barry, Thomas L. | Buffalo Medical College | Feb., 1874. |
| Boyle, Arthur R. | University Queen's College, Kingston, Ont. | April 8, 1859. |
| Coakley, J. B. | Medical College of Virginia | Mar. 1, 1861. |
| Crane, Lucien Darwin | College of Physicians and Surgeons | Feb. 22, 1881. |
| Gregg, Rollin R. | Homœopathic Medical College of Pennsylvania, in Philadelphia | Mar., 1853. |
| Hambleton, R. S., | Hospital College of Medicine, Louisville, Ky. | Feb. 26, 1878. |
| Lansdowne, J. R. | Royal College of Physicians, Edinburgh, and Faculty of Physicians and Surgeons, Glasgow | 1858. |
| Lapp, Henry | Medical Department University of Buffalo | Feb., 1865. |
| Moss, Ransom E. | Buffalo Medical University | Feb. 25, 1880. |
| Mower, J. W. | Albany Medical College | Jan. 27, 1852. |
| Senn, John | College of Physicians and Surgeons | Feb. 23, 1881. |
| Sovereign, Baxter | Hospital College, Cleveland, Ohio | 1869. |
| Van Pelt, William | Regents of the University of the State of New York | Jan. 26, 1841. |
| Weiss, Carl | University of Wurtzburg, Bavaria | June, 1838. |

Sperry, M. M., amends his affidavit by stating that his "authority for practicing is by 23 years' practice in this State, and attending the College of Physicians and Surgeons, at Buffalo, and will graduate in two years, or time specified by law.

